# Supplementary material for: Transcriptome Analysis of Rice Reveals the lncRNA–mRNA Regulatory Network in Response to Rice Black-Streaked Dwarf Virus Infection
Source: Viruses. 2020 Aug 27;12(9):951. doi: 10.3390/v12090951 (PMC7552052; doi:10.3390/v12090951)
Supplement: Supplementary file 1 [file viruses-12-00951-s001.zip › Supplementary Data-Figures.pdf]

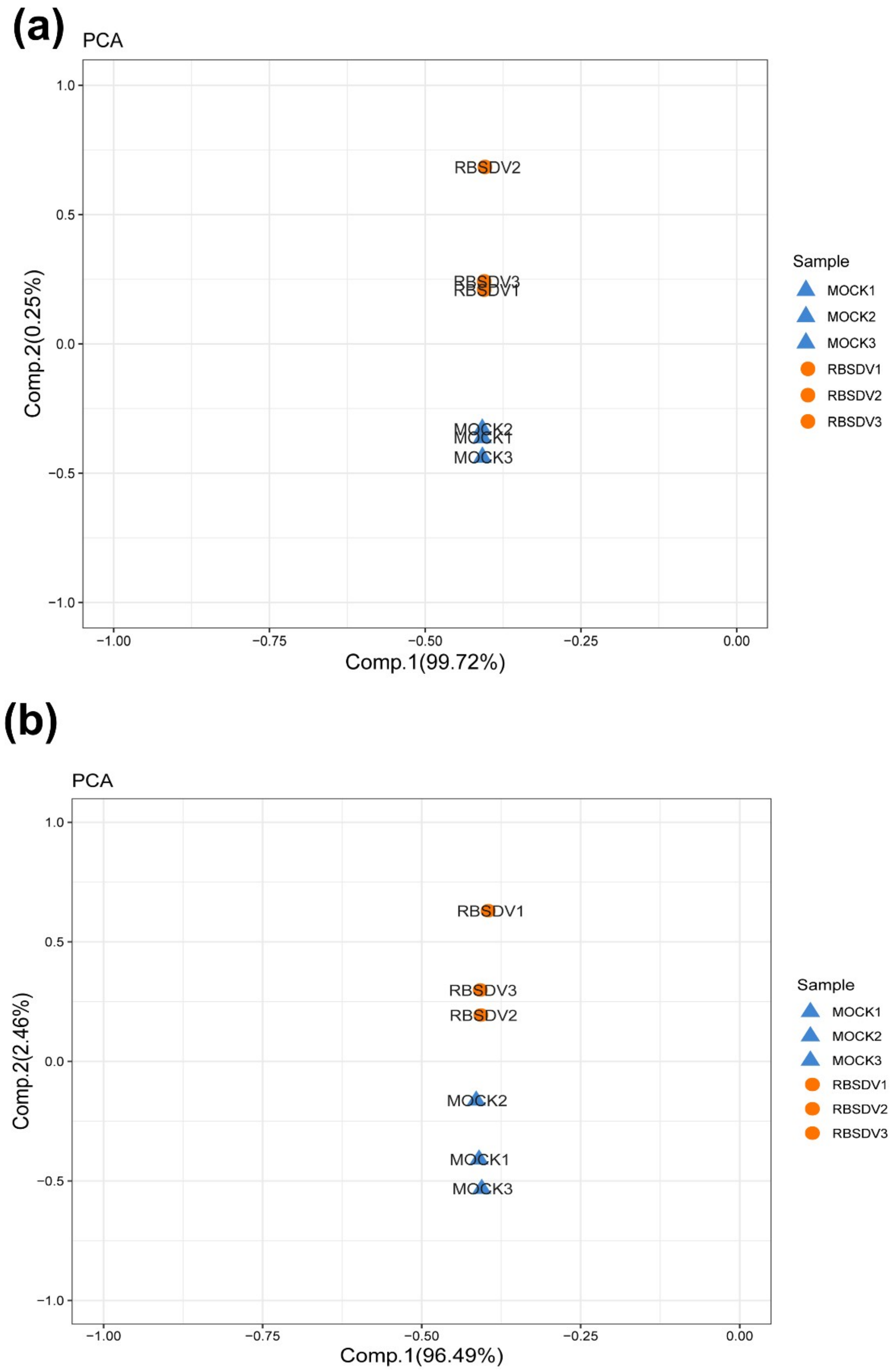

Figure S1. The principal component analysis (PCA) of mRNAs (a) and lncRNAs (b). The yellow circles represent RBSDV-infected rice plants. The blue triangles represent the MOCK rice plants.

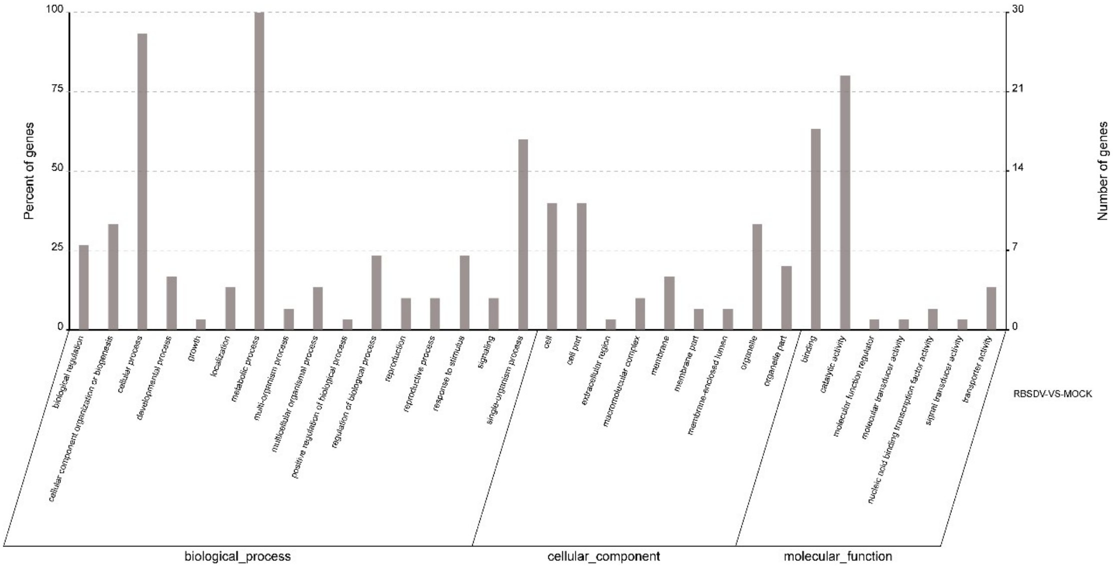

Figure S2. GO enrichment analysis of mRNAs co-located with DElncRNAs identified between the RBSDV-infected and the mock rice plants.

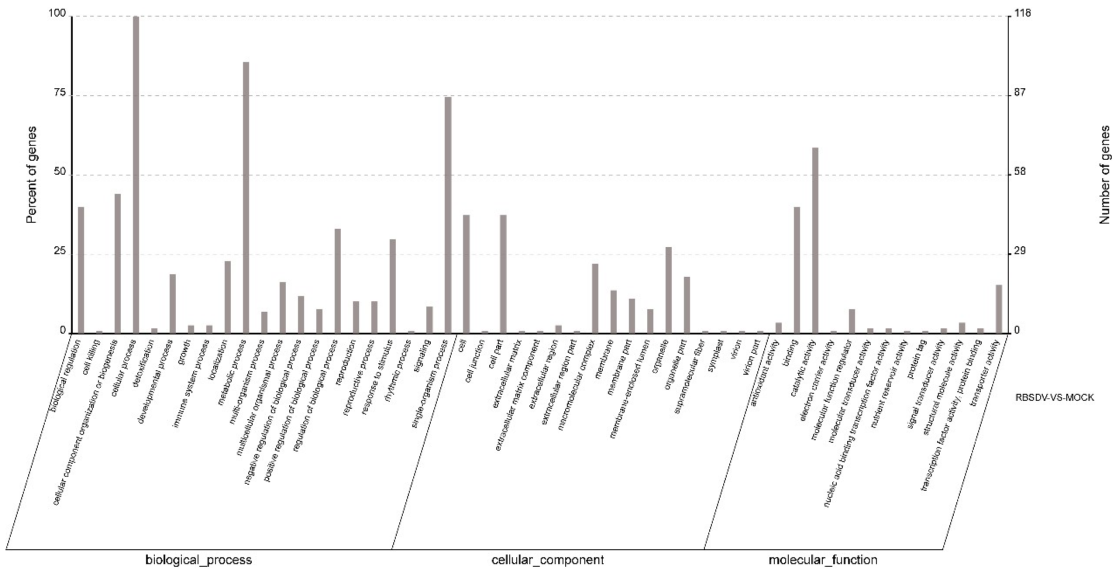

Figure S3. GO enrichment analysis of mRNAs co-expressed with DElncRNAs identified between the RBSDV-infected and the mock rice plants.

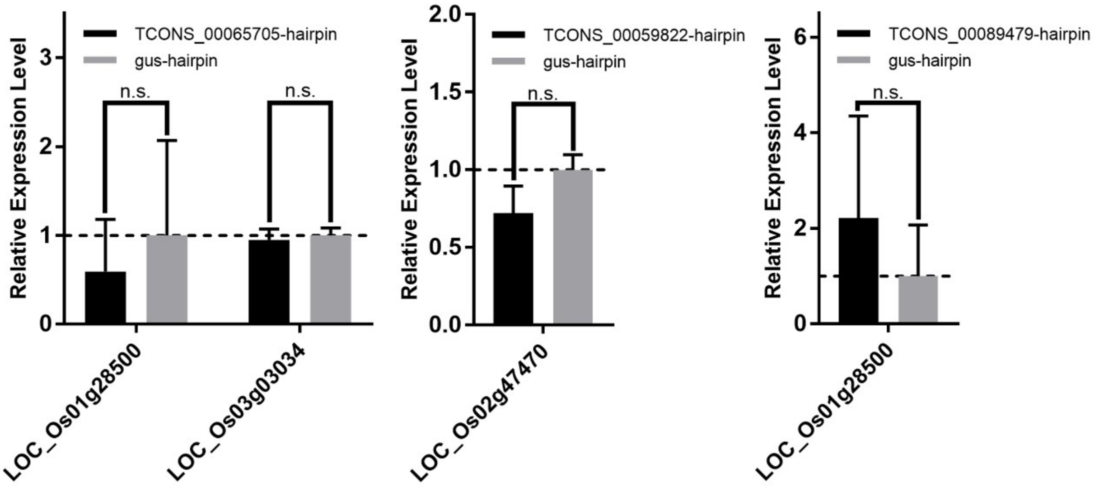

Figure S4. The qRT-PCR data of the mRNAs with no significant changes after silencing lncRNAs. The total RNA of rice callus in Fig. 8 was used for qRT-PCR analyses. *OsUBC* served as an internal reference in relative quantification. The values represent means of the mRNA expression levels ± standard deviations (SD) relative to the gus-hairpin-expressed callus tissues (n = 3 biological replicates). The significant differences between lncRNA-silenced and gus-hairpin-expressed callus tissues were determined by Student's *t*-test.(two-sided, n.s. = not significant)
