# Supplementary material for: Transcriptome Analysis of Rice Reveals the lncRNA–mRNA Regulatory Network in Response to Rice Black-Streaked Dwarf Virus Infection
Source: Viruses. 2020 Aug 27;12(9):951. doi: 10.3390/v12090951 (PMC7552052; doi:10.3390/v12090951)
Supplement: Supplementary file 1 [file viruses-12-00951-s001.zip › Supplementary Data-Tables 1,2,4,7,9.pdf]

Table S1. PCR primer sequences used for qRT-PCR analysis.

Primer-name	Foreword primer	Reverse primer
UBC(LOC_Os02g42314)	CAATCCGTTTGTAGAGCCATAATTGCATTG	GAGGTTGCCTGAGTCACAGTTAAGTG
LOC_Os01g06730	CAATTCCTCCTTGCCTGATCG	CCTCCAATGTGCAACTTTCGT
LOC_Os10g25090	AAGTTTGGCTGCTTCATGCC	TGATCGATATCACTGCAACTAGC
LOC_Os01g28500	TGTTTCACTGCTCCGATGCC	TCCAGCTCATCGGACCCAC
LOC_Os01g56330	AAGTTTTGGCTACCTTGACCC	GGACCTCAAATAGCACAACACC
LOC_Os05g39720	GGCCGTCAATTTGATCAGCAT	AAGTCACAAACCTTACCGGAG
LOC_Os12g02450	TCAATCTGAATTGAGCAGGAGC	TGCACCGTCTTTGTAGCCTT
LOC_Os01g23970	CAATTTGATCTGTGGCAACGAC	CGCTTTCACTCACGATTTCCC
LOC_Os08g42700	AGAATTGCTCCCTCCACACT	TGTTGTCTTTGCCACTGGAAGT
LOC_Os02g03960	AAGCAACTAAGCACTGCACC	TACCATGAGTCCATGAGACAGC
TCONS_00005781	AGCCTTGCCTAGTTAGATTGCAT	TGCGGTCATACCTGTTCGTT
TCONS_00056787	GAGTTTGCCACTTGCTTTTCGT	AATGACTCGGCAACAACCTGA
TCONS_00089479	ACACAAAAGCACAGAGTAGTGA	TCCCAGTCCCTGCTTCAACGA
TCONS_00008316	CTCATAGAGCCACCCTACAGT	ATGTAATTTGCGCCCATCCAC
TCONS_00040997	CGCAAGACATGATTACGACACGA	ACAATTGCCCTAGATAGCTCCT
TCONS_00049628	GCCAGATAATACGCCTCGCA	ACGAGTGGCTCAATGTGCTT
TCONS_00065705_silencing effect detection	ACCCAGAAGAAAGTAAGCCT	TTCACTGGATTCATGCACGAG
TCONS_00089479_silencing effect detection	TCCATGTACTCGAACGTGCCTA	TGTCCAACATCCTGCTCGACT
TCONS_00059822_silencing effect detection	GTACACGCCGAAACCTCGTC	TGAAAGCTTCCCCGTCCCAA
LOC_Os06g44010	CTTGGCTATATATTCCTCCTCGTC	AAGTCTGCTCAATCCACGAATCC
LOC_Os01g06730	CAATTCCTCCTTGCCTGATCG	CCTCCAATGTGCAACTTTCGT
LOC_Os03g18030	TCGCTGGCCTTCTTCTACAACC	TGTGCTCGCGGTACTCGTCGAAG
LOC_Os03g10640	CTATTTGGACTAAAGGGAGAAAATTCCGAT	TGTCTAGAATGCCTTCAAATACATTTATCCTC
LOC_Os04g51660	TCATTGACCGCACGATCATCC	AATCGAACGCATGTTTGGCT
LOC_Os01g28500	TGTTTCACTGCTCCGATGCC	TCCAGCTCATCGGACCCAC
LOC_Os03g03034	TACCGGAACTACACCTACGACGA	GTTGTTCTGAAGAGCTCCAAGCA
LOC_Os02g47470	TGTGGCATTGCTGTCCATCTTCGG	GTTGTACCCCTTCTCCAGCGTCAG

Table S2. PCR primer sequences used for vector construction.

Primer-name	Primer-sequence
1300(BamHI)TCONS89479(exon)-F	AGCTCGGTACCCGGGGATCCGAGCTAGCTACCCTCTACTCA
TCONS89479(exon)+Intron-R	AAAAATCAGACTTACAACGTAGAGCTTCTGACGGGGAAGA
Intron+TCONS89479(exon)-F	TTGGACCCAGCAGATAGAGCAGAGCTTCTGACGGGGAAGA
TCONS89479(exon)(BamHI)1300-R	AGGTCGACTCTAGAGGATCCGAGCTAGCTACCCTCTACTCA
1300(BamHI)TCONS65705(promoter)-F	AGCTCGGTACCCGGGGATCCGGTTCTCAAAGATACAAGTCATG
TCONS65705(promoter)+Intron-R	AAAAATCAGACTTACAACGTCCAAAATTTTATTAATTCCGTGTAT
Intron+TCONS65705(promoter)-F	TTGGACCCAGCAGATAGAGCCCAAAATTTTATTAATTCCGTGTAT
TCONS65705(promoter)(BamHI)1300-R	AGGTCGACTCTAGAGGATCCGGTTCTCAAAGATACAAGTCATG
1300(BamHI)GUS600-F	AGCTCGGTACCCGGGGATCCGTGGTGATGTGGAGTATTGCCA
GUS600(BamHI)1300-R	AGGTCGACTCTAGAGGATCCGTGGTGATGTGGAGTATTGCCA
1300(BamHI)59822(promoter)-F	AGCTCGGTACCCGGGGATCCGACTAGTCGGTCCTGGTTAGG
59822(promoter)+Intron-R	AAAAATCAGACTTACAACGTGTGGCATTTTATTTCCAAAATGG
Intron+59822(promoter)-F	TTGGACCCAGCAGATAGAGCGTGGCATTTTATTTCCAAAATGG
59822(promoter)(BamHI)1300-R	AGGTCGACTCTAGAGGATCCGACTAGTCGGTCCTGGTTAGG

Table S4. KEGG pathway enrichment for DEmRNAs.

Pathway	DEGs with pathway annotation (871)	All genes with pathway annotation (21127)	*p*-value	Pathway ID
Biosynthesis of secondary metabolites	208 (23.88%)	3435 (16.26%)	2.03E-09	ko01110
Metabolic pathways	274 (31.46%)	4974 (23.54%)	3.11E-08	ko01100
Phenylalanine, tyrosine and tryptophan biosynthesis	14 (1.61%)	67 (0.32%)	4.72E-07	ko00400
Amino sugar and nucleotide sugar metabolism	27 (3.1%)	228 (1.08%)	8.78E-07	ko00520
ABC transporters	21 (2.41%)	164 (0.78%)	4.12E-06	ko02010
Stilbenoid, diarylheptanoid and gingerol biosynthesis	31 (3.56%)	329 (1.56%)	1.75E-05	ko00945
Flavonoid biosynthesis	28 (3.21%)	283 (1.34%)	1.82E-05	ko00941
Plant-pathogen interaction	115 (13.2%)	1931 (9.14%)	3.44E-05	ko04626
Plant hormone signal transduction	73 (8.38%)	1120 (5.3%)	7.20E-05	ko04075
Limonene and pinene degradation	22 (2.53%)	224 (1.06%)	1.55E-04	ko00903
Diterpenoid biosynthesis	20 (2.3%)	195 (0.92%)	1.70E-04	ko00904
Glutathione metabolism	16 (1.84%)	139 (0.66%)	2.01E-04	ko00480
Starch and sucrose metabolism	32 (3.67%)	394 (1.86%)	2.20E-04	ko00500
Isoflavonoid biosynthesis	15 (1.72%)	135 (0.64%)	4.60E-04	ko00943
Galactose metabolism	11 (1.26%)	106 (0.5%)	4.32E-03	ko00052
Pyruvate metabolism	11 (1.26%)	108 (0.51%)	4.98E-03	ko00620
Phenylpropanoid biosynthesis	31 (3.56%)	475 (2.25%)	8.23E-03	ko00940
Ascorbate and aldarate metabolism	10 (1.15%)	102 (0.48%)	9.40E-03	ko00053
Carbon fixation in photosynthetic organisms	9 (1.03%)	91 (0.43%)	1.27E-02	ko00710
Steroid biosynthesis	9 (1.03%)	92 (0.44%)	1.36E-02	ko00100

Table S7. KEGG pathway enrichment for mRNAs which are co-located with DElncRNAs.

Pathway	DEGs with pathway annotation (109)	All genes with pathway annotation (21127)	*p*-value	Pathway ID
Indole alkaloid biosynthesis	2 (1.83%)	41 (0.19%)	1.90E-02	ko00901
Lysine degradation	2 (1.83%)	47 (0.22%)	2.45E-02	ko00310
Valine, leucine and isoleucine degradation	2 (1.83%)	56 (0.27%)	3.39E-02	ko00280
Ubiquitin mediated proteolysis	5 (4.59%)	354 (1.68%)	3.66E-02	ko04120
Plant-pathogen interaction	16 (14.68%)	1931 (9.14%)	3.91E-02	ko04626
Pyrimidine metabolism	6 (5.5%)	495 (2.34%)	4.32E-02	ko00240
Non-homologous end-joining	1 (0.92%)	9 (0.04%)	4.55E-02	ko03450
Synthesis and degradation of ketone bodies	1 (0.92%)	9 (0.04%)	4.55E-02	ko00072
Cutin, suberine and wax biosynthesis	3 (2.75%)	163 (0.77%)	5.23E-02	ko00073
Porphyrin and chlorophyll metabolism	2 (1.83%)	76 (0.36%)	5.87E-02	ko00860
Nucleotide excision repair	3 (2.75%)	171 (0.81%)	5.87E-02	ko03420
Alanine, aspartate and glutamate metabolism	2 (1.83%)	83 (0.39%)	6.85E-02	ko00250
Thiamine metabolism	1 (0.92%)	16 (0.08%)	7.94E-02	ko00730
Photosynthesis - antenna proteins	1 (0.92%)	16 (0.08%)	7.94E-02	ko00196
Mismatch repair	2 (1.83%)	92 (0.44%)	8.19E-02	ko03430
Steroid biosynthesis	2 (1.83%)	92 (0.44%)	8.19E-02	ko00100
RNA polymerase	4 (3.67%)	359 (1.7%)	1.15E-01	ko03020
Vitamin B6 metabolism	1 (0.92%)	25 (0.12%)	1.21E-01	ko00750
DNA replication	2 (1.83%)	126 (0.6%)	1.38E-01	ko03030
Selenocompound metabolism	1 (0.92%)	29 (0.14%)	1.39E-01	ko00450

Table S9. KEGG pathway enrichment for mRNAs which are co-expressed with DElncRNAs.

Pathway	DEGs with pathway annotation (2045)	All genes with pathway annotation (21127)	*p*-value	Pathway ID
Biosynthesis of secondary metabolites	410 (20.05%)	3435 (16.26%)	1.07E-06	ko01110
Tryptophan metabolism	25 (1.22%)	122 (0.58%)	2.36E-04	ko00380
Other glycan degradation	29 (1.42%)	155 (0.73%)	4.02E-04	ko00511
Homologous recombination	250 (12.22%)	2155 (10.2%)	1.04E-03	ko03440
Carotenoid biosynthesis	33 (1.61%)	211 (1%)	3.96E-03	ko00906
Plant hormone signal transduction	134 (6.55%)	1120 (5.3%)	5.54E-03	ko04075
Plant-pathogen interaction	219 (10.71%)	1931 (9.14%)	6.12E-03	ko04626
Metabolic pathways	527 (25.77%)	4974 (23.54%)	7.12E-03	ko01100
RNA degradation	183 (8.95%)	1595 (7.55%)	7.58E-03	ko03018
alpha-Linolenic acid metabolism	21 (1.03%)	132 (0.62%)	1.57E-02	ko00592
Glucosinolate biosynthesis	11 (0.54%)	56 (0.27%)	1.71E-02	ko00966
Glycosphingolipid biosynthesis - ganglio series	6 (0.29%)	23 (0.11%)	1.95E-02	ko00604
Amino sugar and nucleotide sugar metabolism	32 (1.56%)	228 (1.08%)	2.08E-02	ko00520
Flavonoid biosynthesis	38 (1.86%)	283 (1.34%)	2.42E-02	ko00941
Zeatin biosynthesis	127 (6.21%)	1108 (5.24%)	2.42E-02	ko00908
Phenylpropanoid biosynthesis	59 (2.89%)	475 (2.25%)	2.78E-02	ko00940
Ribosome biogenesis in eukaryotes	176 (8.61%)	1593 (7.54%)	3.19E-02	ko03008
Phenylalanine metabolism	30 (1.47%)	222 (1.05%)	3.84E-02	ko00360
Glycosaminoglycan degradation	7 (0.34%)	35 (0.17%)	4.75E-02	ko00531
Galactose metabolism	16 (0.78%)	106 (0.5%)	4.87E-02	ko00052
